# Local delivery of Doxorubicin and Olaparib loaded injectable hydrogels with adjuvant radiotherapy improves survival in a glioblastoma in vivo model

**DOI:** 10.1007/s13346-025-02003-7

**Published:** 2025-10-23

**Authors:** Robert Cavanagh, Gary Shaw, Phoebe McCrorie, Amr ElSherbenny, Alina Pandele, Supisara Jearranaiprepame, Bayan Ghanem, Natalie Allcock, Heiko Wurdak, Ryan K. Mathew, Cameron Alexander, Ruman Rahman, Cara Moloney

**Affiliations:** 1 School of Pharmacy, University of Nottingham, Nottingham, NG7 2RD UK; 2 Leeds Institute of Medical Research, School of Medicine, University of Leeds, Leeds, LS9 7TF UK; 3 School of Medicine, Biodiscovery Institute, University of Nottingham, Nottingham, NG7 2RD UK; 4 University of Petra Pharmaceutical Centre, University of Petra, 11196 Petra, Jordan; 5 Electron Microscopy Facility, University of Leicester, Leicester, LE1 7HB UK; 6 Department of Neurosurgery, Leeds Teaching Hospitals NHS Trust, Leeds, LS1 3EX UK

**Keywords:** Local drug delivery, Glioblastoma, Polymer hydrogel, Doxorubicin, Olaparib, Radiotherapy

## Abstract

Local drug delivery systems (LDDS) are a promising method to overcome challenges associated with chemotherapeutic treatment of brain tumours, namely poor blood-brain barrier penetration. Here we report a poly(ethyleneglycol)-poly(lactide)-poly(caprolactone)-poly(lactide)-poly(ethyleneglycol) based injectable hydrogel, PELCLE, loaded with Doxorubicin (Dox) and Olaparib (Ola) as an LDDS against glioblastoma (GBM), a primary malignant brain tumour with a poor prognosis. The thermoresponsive properties of the hydrogel, which behaved as a liquid at room temperature and formed a gel at elevated temperatures, were not impacted by the inclusion of chemotherapeutics whereby two-week sustained release was recorded for both Dox and Ola. Drug potency was assessed against a panel of GBM cell lines, both a syngeneic mouse line and primary patient-derived lines, and the combination of Dox/Ola demonstrated synergistic effects at a range of drug: drug ratios. The application of radiotherapy (XRT) in combination with Dox/Ola improved treatment efficacy both in vitro and in vivo, with a significant increase in median survival observed when Dox/Ola PELCLE hydrogels were applied against a surgical resection model of GBM (syngeneic mouse model SB28) with and without the addition of adjuvant XRT (28 and 23 days, respectively, *p* < 0.01). Furthermore, a long-term survivor was noted in the group treated with the drug loaded HG and XRT, which was associated with a very small residual tumour, indicating the efficacy of this treatment against a GBM in vivo model.

**Supplementary Information:**

The online version contains supplementary material available at 10.1007/s13346-025-02003-7.

## Graphical abstract

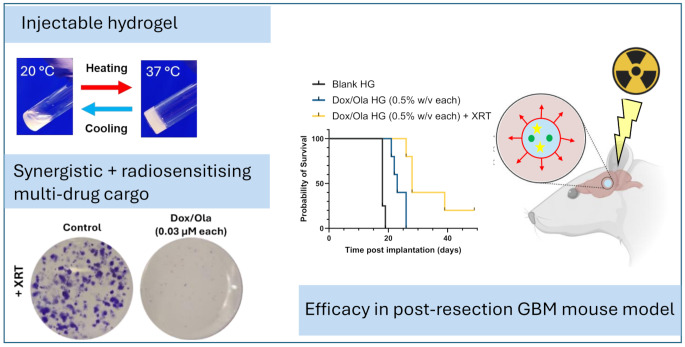

## Introduction

Glioblastoma (GBM) is the most prevalent and malignant brain tumour and despite extensive research, median survival remains low, at 15 months from time of diagnosis [1]. Current standard-of-care consists of surgical resection of the tumour bulk, followed by concomitant oral temozolomide (TMZ) and radiotherapy (XRT) [2]. However, patients exhibit varying responses to TMZ, with those that have unmethylated O6-methylguanine-DNA-methyltransferase (MGMT) gene promoters exhibiting poorer responses [3]. As such, there is a need to identify additional drugs (and drug combinations) which have broader applicability across the patient range that could be applied in addition to TMZ to improve patient outcomes, but this is limited by the restrictive nature of the blood-brain barrier (BBB), which permits only drugs with specific physical properties, such as size and lipophilicity, to cross [4–6].

To address this clinical need, local drug delivery systems (LDDS) have gained much attention to improve the penetration of drugs across the BBB. The technique involves the application a drug depot directly into the tumour resection cavity following surgical debulking, bypassing the BBB completely. Furthermore, as there is typically a delay of up to 6 weeks between surgical resection and adjuvant therapies [7], the application of a LDDS during surgery offers a means to treat residual tumour cells within this gap, potentially preventing or delaying tumour recurrence. To date, there has been only one clinically approved LDDS for the treatment of GBM, Gliadel^®^, which is composed of solid polymer discs loaded with carmustine that are placed in contact with the parenchyma [8]. Limitations of this system stem from the inability of the wafers to adhere to the surrounding parenchyma and the mismatch in stiffness between the wafers and the brain which can result in adverse side effects [9]. Drug loaded hydrogels (HGs) show great promise as LDDS [10] as their injectability allows for conformation within the post-surgical cavity which is often irregularly shaped [11]. Injectable hydrogels have been widely reported in the literature for treatment of GBM and can be prepared from a variety of materials, including a range of polymers [12], peptides [13] and supramolecular systems [14].

The topoisomerase II inhibitor Doxorubicin (Dox) has demonstrated efficacy against GBM models in vivo [15] and LDDS loaded with Dox have been developed [16, 17]. Wang et al. reported Dox prodrug nanoparticle loaded hydrogels (HGs) against an orthotopic GBM resection model and demonstrated that they were well tolerated, exhibited sustained Dox release and enhanced survival compared to untreated controls [18]. Similarly, we have recently shown local delivery of Olaparib (Ola) to be efficacious against GBM models in vivo [19], with enhanced survival observed when adjuvant XRT, etoposide or temozolomide was also applied. Due to the capabilities of Ola as a radiosensitiser [20], it has also been investigated in a Phase I clinical trial where it was administered concurrently with XRT and shown to be well tolerated by patients [21]. Collectively, this warrants consideration of Dox/Ola combination for GBM, predicated on potential DNA damage inducing/sensitising effects respectively.

Furthermore, anti-cancer drug combinations present as a promising strategy to overcome tumour heterogeneity and drug resistance, coupled with enhanced efficacy when drug synergism can be achieved [22]. Dox/Ola combinations have been shown to be effective in vitro, with synergism noted against triple negative breast cancer [23] and ovarian cancer [24] cell lines. The enhanced cytotoxic effect is reportedly due to the combination of the DNA damaging capabilities of Dox [25] with the PARP1 inhibitory capabilities of Ola which prevents PARP-mediated DNA damage repair [26], leading to increased levels of apoptosis [23]. The application of PEGylated polypeptide nanogels loaded with Dox/Ola against an in vivo triple negative breast cancer model resulted in a significant reduction in tumour growth as compared to untreated controls and free Dox/Ola treatments, improved inhibition of proliferation of tumour cells, and an enhancement in induced apoptosis [27]. Furthermore, the combination of Ola with PEGylated liposomal Dox was found to be effective against platinum resistant ovarian cancer in a Phase II clinical trial [28]. While research has been undertaken using Dox/Ola combinations against TNBC and ovarian cancers, to the best of our knowledge, there has been no report of the combination of Dox and Ola in the treatment of GBM.

In this work, we report on the preparation and characterisation of a poly(ethyleneglycol)-poly(lactide)-poly(caprolactone)-poly(lactide)-poly(ethyleneglycol) based HG, mPEG-PLA-PCL-HMDI-PCL-PLA-mPEG, (or PELCLE), which is injectable and can be applied as an LDDS. The HG was loaded with a dual combination of Dox and Ola and the release over two weeks was quantified, commensurate with the clinical oncological treatment gap between surgery and TMZ/XRT. The cytotoxicity of Dox and Ola at a range of combination ratios were investigated against a panel of GBM cell lines to evaluate synergistic effects. The impact of adjuvant radiation with drug treatments was evaluated in vitro using a clonogenic assay. In vivo studies were undertaken in a tumour resection model of the syngeneic GBM cell line, SB28-Ohlfest [29], to investigate: (i) the feasibility of PELCLE HG application intraoperatively; (ii) the efficacy of Dox/Ola loaded PELCLE HG as compared to blank HG; and (iii) the impact of adjuvant XRT with the drug loaded HG on overall survival. Histological analyses were undertaken on the brains at the end of the study to evaluate tissue level effects.

## Methods

### Preparation and characterisation of mPEG-PLA-PCL-HMDI-PCL-PLA-mPEG (PELCLE)

The multi-block co-polymer mPEG-PLA-PCL-HMDI-PCL-PLA-mPEG (PELCLE) was prepared as previously reported [30]. In brief, mPEG (5 g, 0.01 mmol, 500 g mol^− 1^) was azeotropically distilled in anhydrous toluene at 50 °C. Following this, D,L-lactide (5.8 g, 0.04 mol) was added and the reaction mixture was stirred under a nitrogen atmosphere at 120 °C. Once homogenised, Sn(Oct)_2_ (52.2 mg, 0.0004 mmol) dissolved in dichloromethane was added and the reaction was left stirring overnight, resulting in mPEG-PCL diblock polymer with complete monomer conversion as demonstrated by ^1^H NMR. To the mPEG-PCL diblock polymer, ε-caprolactone (4.3 g, 0.038 mol) was added and the reaction mixture was stirred under a nitrogen atmosphere at 120 °C overnight, with ^1^H NMR employed to confirm successful synthesis of the tri-block polymer. Finally, the reaction mixture was cooled to 80 °C, HMDI (1.68 g, 0.01 mmol) was added, and the reaction was allowed to proceed for a further 24 h. The resulting product was dissolved in dichloromethane, precipitated in ice cold hexane and dried in a vacuum oven overnight before characterising with ^1^H NMR. Full assignments of the ^1^H NMR spectra of the product employed in this work, along with GPC and FT-IR characterisation have been previously reported [30]. In brief, M_n_ was calculated to be 3230 g mol^− 1^ by ^1^H NMR and M_w_ was determined to be 13,240 g mol^− 1^, with a polydispersity index of 1.1, by GPC analysis.

### Preparation and characterisation of PELCLE hydrogel

PELCLE hydrogels (HGs) were prepared at 30% (w/v) by dissolving the polymer in PBS using repeated cycles of heating (50 °C), vortexing, and cooling (4 °C). The HG was able to be stored at 4 °C without visually observable changes to its ability to flow. Drug loaded HGs for rheological, release and in vivo studies were prepared by dissolving Dox, Ola or a combination in PELCLE HGs at a concentration of 0.5% (w/v) for each drug.

The rheological behaviour of PELCLE HGs, with and without the incorporation of drugs, was investigated using an Anton Paar MCR 302 Modular Compact Rheometer (Austria). HGs were prepared at PELCLE concentrations of 30% (w/v), and included blank HG, and Dox/Ola loaded HG at 0.5% (w/v) of each drug. The samples were placed between parallel plates (25 mm diameter, 1 mm gap) and the impact of temperature on the storage (G’) and loss modulus (G”) was measured between 10–60°C at a heating rate of 1°C/min under controlled strain (1%) and frequency (1.0 Hz). Recorded data was analysed using RheoCompass software (Austria) and the gelation temperature (T_gel_) was determined as the point at which G’ surpassed G”.

### In vitro drug release

The release profiles of Dox and Ola from PELCLE HGs were recorded at 37 °C in PBS at pH = 7.4, i.e. physiological pH. 50 µL of drug loaded PELCLE (single or dual agent at loading concentrations of 0.5% (w/v) of each drug) was pipetted into a 1.5 mL centrifuge tube and set at 37 °C for 30 min before incubating in 1 mL PBS. At desired time intervals (4 h, 1-, 3-, 7- and 14-day timepoints), the PBS was removed and replaced with a fresh aliquot to ensure that sink conditions are maintained. HPLC analysis (Agilent 1200) was employed to quantify the drug content in the release media using a mobile phase gradient of 10 mM sodium phosphate buffer (pH = 6.7) and ACN at a flow rate of 1 mL/min. ACN content was maintained at 20% for 5 min followed by a linear increase to 60% over 15 min, before returning to the original conditions over 2 min. An ACE C18 column (4.6 × 250 mm, 5 μm) was used with a VWD set to 254 nm for detection. 50 µL of sample was injected and a run time of 30 min was used.

### In vitro cell culture and drug IC50 assessment

GCE28 and GIN28 cells primary GBM cell lines were isolated from the 5-ALA fluorescence-positive core and invasive margin, respectively, during patient surgery (71 y male, wild-type IDH (primary GBM), intact ATRX, 0% MGMT promoter methylation 99% resection; no adjuvant therapy (patient choice); died 3 months after surgery) [14]. The cells were routinely maintained in DMEM with 10% foetal bovine serum at 37 °C in 5% CO_2_-humidified incubators. The SB28-Ohlfest cell line (obtained from the DSMZ Leinniz Institute, Germany, and established by Ohlfest et al. in a murine model [29]) was maintained in high glucose DMEM with 10% foetal bovine serum. The PrestoBlue™ Cell Viability assay was performed to assess metabolic activity of cells following exposure to Dox, Ola and a dual treatment at varying ratios.

For IC50 and synergy studies, cells were seeded at a density of 5 × 10^3^ cells per well and incubated overnight. Following this, the media was removed, replaced with drug treatments diluted in media or a negative control (media only) and incubated for 72 h. Following exposure, the media was removed and replaced with 100 µL of 10% (v/v) PrestoBlue™ reagent diluted in PBS per well and the plate incubated for 30 min at 37 °C. Fluorescence was measured at 544/590 nm (ex/em) on a TECAN Infinite M Plex plate reader. Relative metabolic activity was calculated by setting normalised values from the negative control as 100%. IC50 values were calculated using GraphPad prism and dose response curves.

For evaluation of synergy between Dox and Ola drug combinations, IC50 values were determined, as described above, for (i) Dox alone; (ii) Ola alone; and (iii) combinations of Dox and Ola at varying ratios (100:1, 10:1, 1:1, 1:10 and 1:100 Dox: Ola). The Chou and Talay method [31, 32] was then employed to calculate combination index (CI) values, where D_SD_ is the IC50 value of Dox alone, D_SO_ is the IC50 value of Ola alone, D_CD_ is the IC50 value of Dox in combination with Ola, and D_CO_ is the IC50 value of Ola in combination with Dox.

$$\displaylines{{\mathrm{CI}} = \left( {{{{{\mathrm{D}}_{{\mathrm{CD}}}}} \mathord{\left/{\vphantom {{{{\mathrm{D}}_{{\mathrm{CD}}}}} {{{\mathrm{D}}_{{\mathrm{SD}}}}}}} \right.\kern-\nulldelimiterspace} {{{\mathrm{D}}_{{\mathrm{SD}}}}}}} \right) + \left( {{{{{\mathrm{D}}_{{\mathrm{CO}}}}} \mathord{\left/{\vphantom {{{{\mathrm{D}}_{{\mathrm{CO}}}}} {{{\mathrm{D}}_{{\mathrm{SO}}}}}}} \right.\kern-\nulldelimiterspace} {{{\mathrm{D}}_{{\mathrm{SO}}}}}}} \right) \cr + \left( {{{\left( {{{\mathrm{D}}_{{\mathrm{CD}}}}*{{\mathrm{D}}_{{\mathrm{CO}}}}} \right)} \mathord{\left/{\vphantom {{\left( {{{\mathrm{D}}_{{\mathrm{CD}}}}*{{\mathrm{D}}_{{\mathrm{CO}}}}} \right)} {\left( {{{\mathrm{D}}_{{\mathrm{SD}}}}*{{\mathrm{D}}_{{\mathrm{SO}}}}} \right)}}} \right.\kern-\nulldelimiterspace} {\left( {{{\mathrm{D}}_{{\mathrm{SD}}}}*{{\mathrm{D}}_{{\mathrm{SO}}}}} \right)}}} \right) \cr} $$

CI values < 0.9 were deemed synergistic, values between 0.9 and 1.1 were deemed additive and values > 1.1 were deemed antagonistic.

### Clonogenic assays

The impact of Dox and/or Ola in combination with radiation in vitro was investigated. Briefly, cells were seeded at a density of 2 × 10^5^ cells in T25 flasks and incubated for 24 h. Following this, the media was removed, replaced with drug treatments diluted in media (Dox/Ola at 0.03 µM each, Dox at 0.03 µM, Ola at 0.03 µM or Ola at 4.6 µM) or media only for controls, and incubated for 24 h. Flasks were then irradiated using an Xstrahl RS320 X-ray Irradiator at 2 Gy and incubated for 24 h. 2 Gy was chosen as this was shown in preliminary scoping experiments to impact the clonogenicity of the cells post-treatment at a level that was quantifiable. Cells were counted and seeded in 6-well plates at low densities (0.3–4 × 10^3^ cells/well for controls, 0.5–10 × 10^3^ cells/well for treatment groups) for colony formation studies using fresh media. After 5 days of growth, colonies were fixed using 10% formalin, stained with 0.5% crystal violet and colonies containing 50 or more cells were counted. All treatment conditions (control ± 2 Gy, Dox/Ola at 0.03 µM each ± 2 Gy, Dox at 0.03 µM ± 2 Gy, Ola at 0.03 µM ± 2 Gy or Ola at 4.6 µM ± 2 Gy) were repeated in triplicate. The number of colonies formed were counted and the plating efficiency (PE) was calculated for the untreated cells, while the surviving fraction (SF) was calculated for the drug and/or XRT treated cells, using the equations below [33]. The SFs have been reported as mean ± SEM.

$$\displaylines{{\text{PE = }} \cr {{{\mathrm{no}}{\mathrm{.}}\,{\mathrm{of}}\,{\mathrm{colonies}}} \mathord{\left/{\vphantom {{{\mathrm{no}}{\mathrm{.}}\,{\mathrm{of}}\,{\mathrm{colonies}}} {{\mathrm{no}}{\mathrm{.}}\,{\mathrm{of}}\,{\mathrm{cells}}\,{\mathrm{seeded}}}}} \right.\kern-\nulldelimiterspace} {{\mathrm{no}}{\mathrm{.}}\,{\mathrm{of}}\,{\mathrm{cells}}\,{\mathrm{seeded}}}} \cr} $$

$$\displaylines{{\text{SF = }} \cr {{{\mathrm{no}}{\mathrm{.}}\,{\mathrm{of}}\,{\mathrm{colonies}}\,{\mathrm{formed}}} \mathord{\left/{\vphantom {{{\mathrm{no}}{\mathrm{.}}\,{\mathrm{of}}\,{\mathrm{colonies}}\,{\mathrm{formed}}} {\left( {{\mathrm{no}}{\mathrm{.}}\,{\mathrm{of}}\,{\mathrm{cells}}\,{\mathrm{seeded}}\, \times \,{\mathrm{PE}}} \right)}}} \right.\kern-\nulldelimiterspace} {\left( {{\mathrm{no}}{\mathrm{.}}\,{\mathrm{of}}\,{\mathrm{cells}}\,{\mathrm{seeded}}\, \times \,{\mathrm{PE}}} \right)}} \cr} $$

### In vivo studies

In vivo studies were carried out under the UK Home Office licence PP6917196 fully compliant with the guiding principles for the care and use of laboratory animals in the UK. Male and female C57BL/6J mice were bred in house and used at an age of 12 to 16 weeks. They were maintained in groups of five in individually ventilated cages containing sawdust, paper bedding and environmental enrichment. Mice were housed at 20 ± 2 °C under a 12-hour light/12-hour dark photoperiod and received standard rodent pelleted chow *ad libitum* (Special Diets Services, Witham, UK).

For tumour implantation, animals were placed on a stereotaxic frame (Stoelting), anaesthetised with isoflurane and 1.5 × 10^4^ SB28-Ohlfest cells in 2 µl of serum free DMEM were injected at co-ordinates (from bregma) + 2.00 mm anterior posterior, + 1.50 medial lateral and − 3.00 mm dorsal ventral. Mice were monitored daily and scored according to weight loss and symptoms. On day 7 post implantation, tumour growth was confirmed by IVIS imaging. Mice were injected subcutaneously with luciferin (2 µg per mouse), anaesthetised with isoflurane and scanned with an IVIS Lumina (Caliper Life Sciences). Living Image software (Caliper Life Sciences) was used to obtain the maximum radiance over each region of interest relative to negative control. Mice underwent a craniotomy on day 13 to surgically resect the tumours. Mice were anaesthetised using 3% (v/v) isoflurane and transferred to a stereotactic frame. The skin was disinfected, an incision was made and the skull surface allowed to dry to locate the bregma and to identify the co-ordinates used for inoculation. A high-speed drill (Ideal 60-1000) with a round 1 mm burr was used to expand the area of injection and create the craniotomy. A heated fine needle (31G) and forceps were used to remove the tumours and the cavities were irrigated with sterile 0.9% NaCl solution. Celstat (Baster Biosurgery) was temporarily placed into the cavity to achieve haemostasis. Following this, approximately 30 µL HG with or without drugs was injected into the cavity. The following treatment groups were included in the study: (1) blank PELCLE HG at 30% (w/v) (*n* = 4); (2) Dox/Ola loaded PELCLE HG at 0.5% (w/v) each (equivalent to 0.15 mg of each drug per 30 µL dose) (*n* = 5); and (3) Dox/Ola loaded PELCLE HG at 0.5% (w/v) each with the addition of radiation therapy. Radiation therapy at 5 Gy was administered on day 18, 19 and 20 (using a Small Animal Radiation Research Platform SARRP (Xstrahl). This dose was chosen as it was calculated to be a biologically effective dose [34]. The skin was closed using 5−0 prolene sutures (Ethicon) and surgical glue (Gluture). During the procedure mice received baytril antibiotic (Baxter) and painkillers, meloxicam (Boehringer Ingelheim) and bupivacaine (Aspen). Mice were removed from the frame and allowed to recover in a warming chamber. A flavoured electrolyte replenisher gel was given to provide nutrients, hydration and accelerate recovery (LBS-Biotech). Mice were monitored closely for 5 days following the procedure, given painkillers (meloxicam) as required. A structured scoring system was employed to monitor animal welfare throughout the study where individual parameters such as grooming, posture, activity, breathing, orientation, and social interaction were each assigned weighted scores reflecting the severity of deviation from normal behaviour. These were summed to provide an overall welfare score, and animals showing rapid deterioration or reaching the predefined threshold were immediately removed from the study to prevent further suffering. Importantly, death is not permitted as an endpoint under a UK Home Office licence; humane intervention occurs before this stage. Any animals failing to recover fully from craniotomy within 72 h were excluded from the experiment. At end points of the study, mice were humanely culled via overdose of pentobarbital, brains were harvested and fixed in 10% formalin. Animals alive at 49 days post tumour implantation were deemed long-term survivors (LTS) as they survived twice as long as the last in the control group.

### Immunohistochemistry

A tissue processor (Leica TP1020) was employed to embed the fixed post-mortem brains in paraffin prior to sectioning to a thickness of 10 μm on a microtome (Leica RM2245). Haematoxylin and Eosin (H&E) staining was conducted to assess the tissue level effects following treatments of the longest survivor in each group. In brief, paraffin embedded sections were submerged in xylene for deparaffinisation, rehydrated through sequential submersion in 100, 90, 70, 50% IMS and water, and stained with Harris haematoxylin and eosin (Surgipath, UK). The sections were then dehydrated and mounted with DPX mounting medium (Sigma) before imaging on a NanoZoomer^®^-SQ (Hamamatsu).

### Statistical analysis

In vitro cytotoxicity results are reported as the inhibitory concentration 50% (IC50) for each cell line given as the mean and standard error of the mean (SEM) for three independent experiments, plotted relative to the percent viability of vehicle-normalized untreated cells. Calculated survival fraction from clonogenic survival assays were reported as the mean and SEM for three independent biological replicates. Statistical analyses for both experiments were carried out using a two-way ANOVA with Tukey’s multiple comparisons, with differences considered significant when **** *p* < 0.0001, *** *p* < 0.001, ** *p* < 0.01, * *p* < 0.05.

Overall survival (OS) analyses were performed using GraphPad Prism (v10.2). OS was calculated from time of tumour implantation to death from any cause. Kaplan–Meier survival curves with significance levels determined by the log-rank test were constructed by univariate analyses. *P* values < 0.05 were deemed statistically significant.

## Results and discussion

**Fig. 1:**
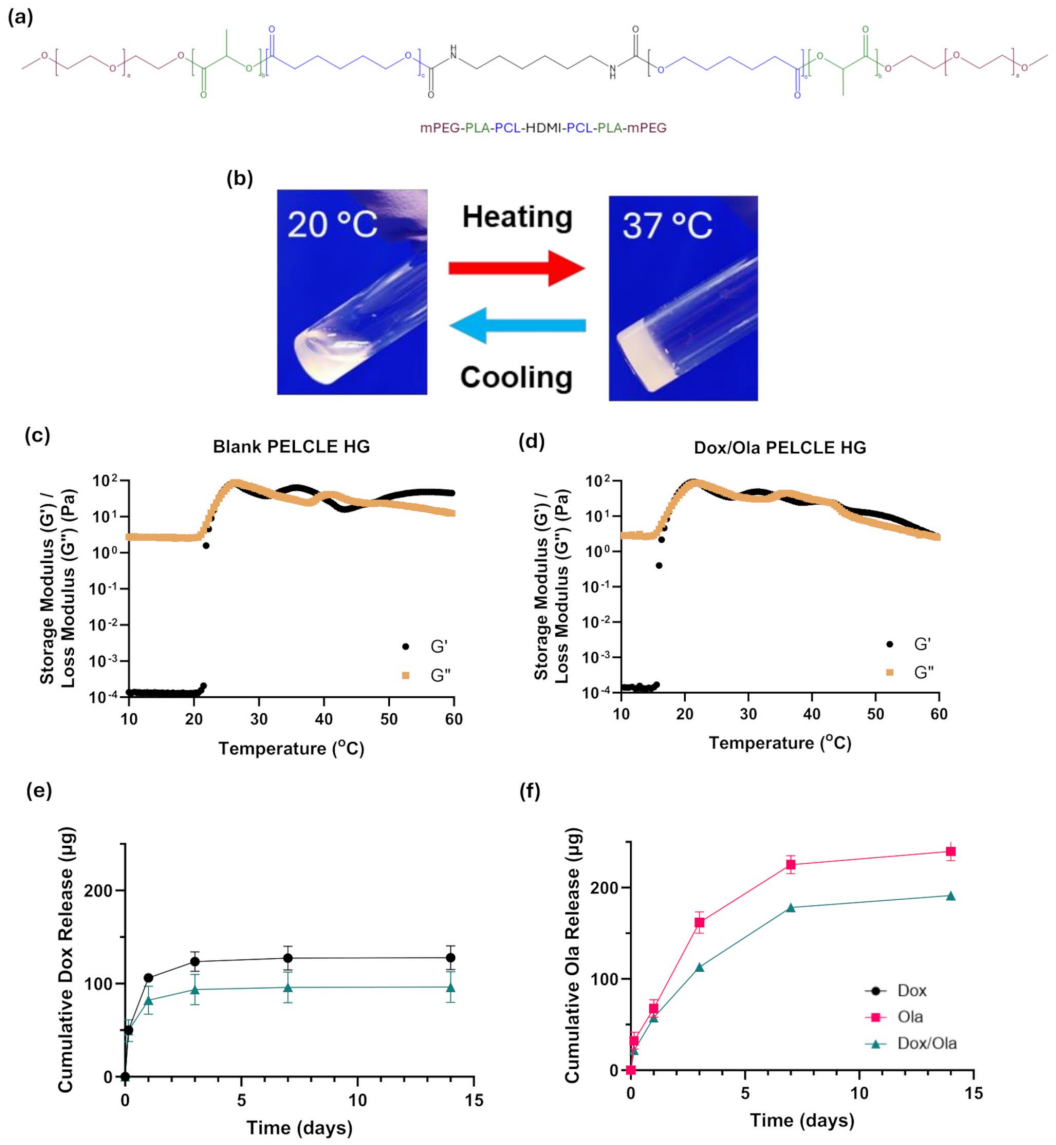
PELCLE hydrogel characterisation and in vitro release (**a**) Schematic representation of the chemical structure of PELCLE. (**b**) Visual representation of PELCLE HG at 30% (w/v) setting at 37 °C. Rheological analysis of (**c**) blank and (**d**) drug loaded HG at 30% (w/v) and Dox/Ola loadings of 0.5% (w/v) each. (**e**) and (**f**) Cumulative amounts of Dox and Ola release, respectively, from PELCLE HG prepared at 30% (w/v) and loaded with Dox, Ola or a combination at loadings of 0.5% (w/v) of each drug (*n* = 3)

The multi-block co-polymer mPEG-PLA-PCL-HMDI-PCL-PLA-mPEG (PELCLE) (Fig. 1a) was chosen for application as a local drug delivery system (LDDS) for brain tumours in this work. We have previously reported this system as a LDDS, that it is injectable through a 29G needle, against pancreatic cancer [30]. We demonstrated that the HG could be injected intra-tumourally, retained in situ at the injection site and did not alter the tissue architecture or increase the levels of apoptosis or proliferation within the tumour as compared to a saline control, indicating the suitability of this system as biocompatible drug depot [30]. The PELCLE polymer was accordingly synthesised as previously described [30], via ring-opening polymerisation of caprolactone followed by coupling through a diisocyanate. The full characterisation data for the PELCLE polymers and pre-cursors has been previously reported by us [30], with an adapted and annotated version of the ^1^H NMR spectra reported in Figure S1 for ease of reference and to highlight the key transformations along the synthetic pathway.

The thermoresponsive properties of PELCLE HG prepared at 30% (w/v) were investigated using a test-tube-inversion method (Fig. 1b), where the sample behaved as a free-flowing solution at 20°C but underwent at sol-gel transition when heated to 37°C. This observation is supported by dynamic rheological experiments (Fig. 1c) which show that below 20°C, both the storage (G’) and loss (G”) modulus are low (< 3 Pa), confirming the free-flowing state observed visually at this temperature. The gelation point (T_gel_), the point at which the HG has become physically crosslinked, was identified as the temperature at which G’ >G”, and occurred at approximately 33 °C. This is a desired property of an injectable LDDS as it (i) allows for the system to pass through a syringe, or similar injection device, at room temperature, allowing for precise control over the dose administered; and (ii) results in the system gelling once injected at the desired location in the body due to the T_gel_ being lower than body temperature.

Another desired property to consider when designing a LDDS is the stiffness of the material, as the application of a drug depot with a higher stiffness than that of the brain parenchyma can result in adverse side effects [9]. At 37 °C, a stiffness value of 0.1 kPa was recorded at 60 Hz for a PELCLE HG prepared at 30% (w/v). This is lower than that reported for the stiffness of the brain under similar rheological conditions, 1–3.5 kPa at 37 °C and a frequency of 50–60 Hz [35], indicating the potential of our LDDS to circumvent the side effects noted following the application of other LDDS.

When the chosen chemotherapeutics for this work, a combination of Dox and Ola, were incorporated into the PELCLE HG at loading concentrations of 0.5% (w/v) of each drug (Fig. 1d), the system again behaved as a free-flowing solution at low temperatures, but began to gel as temperature was increased, with a T_gel_ of approximately 29 °C. The small shift in T_gel_ following drug loading is likely due to the drug interaction with the micellar cross-linking process within the HG as the temperature is increased, a phenomenon which was previously reported when celecoxib was loaded into HGs with a similar structure (P(CL-co-LA)-PEG-P(CL-co-LA)) [36]. Nonetheless, the drug loaded HG possesses the desired properties of an injectable LDDS as the recorded T_gel_ is greater than room temperature, thus allowing for passage through a syringe or similar device, and the integrity of the crosslinked system is retained at 37 °C.

An issue associated with Gliadel^®^ wafers for the treatment of GBM arises from the lack of adherence of the polymer discs to brain parenchyma due to the uneven nature of the cavity following tumour resection. This is exacerbated in the recovery room or upon extubation when patients frequently cough, causing transient spikes in intracranial pressure that cause the cavity to mechanically deform and further disrupt brain-disc contact. The injectability of our system offers a mean to overcome this as the LDDS is introduced as a liquid which fills the cavity before gelling due to the elevated temperature of the body. As a result, the gel conforms to the shape of the cavity, achieving better contact with the surrounding parenchyma harbouring residual disease, enabling delivery of loaded chemotherapeutics in proximity.

The release of Dox and Ola, both as single and dual loaded chemotherapeutics, was quantified from the PELCLE HG when loaded at 0.5% (w/v) of each drug for up to two weeks with incubation in PBS (pH = 7.4) at 37 °C (Fig. 1e and f, S2). Dox loaded HGs exhibited a burst release within the first 72 h or incubation (123.6 ± 10.5 µg, or 49.4 ± 4.2%), followed by low levels of release up to 2 weeks of incubation (127.9 ± 12.7 µg, or 51.1 ± 5.1%). The release of Dox from the dual loaded HG was slightly reduced, with 93.6 ± 16.3 µg (37.4 ± 6.5%) released within the first 72 h and 96.4 ± 16.4 µg (38.5 ± 6.6%) released following 2 weeks of incubation. Ola release followed a more sustained profile, with 161.7 ± 11.7 µg (64.1 ± 4.6%) released within the first 72 h of incubation and almost complete release observed following 2 weeks of incubation, 239.8 ± 10.3 µg (95.0 ± 4.1%). The inclusion of Dox as a combination drug formulation again reduced the levels of Ola released, 113.0 ± 2.0 µg (44.8 ± 0.8%) following 72 h and 191.3 ± 1.9 µg (75.8 ± 0.7%) following 2 weeks.

The current standard of care for GBM treatment follows the Stupp Protocol which involves surgical resection of the tumour, followed by administration of oral temozolomide (TMZ) with adjuvant radiotherapy (XRT) [2]. The delay between surgery and administration of TMZ and XRT typically ranges between 2 and 6 weeks [7] and during this time residual cancer cells can proliferate, leading to tumour recurrence. The sustained release profiles up to 2 weeks of Dox and Ola from our LDDS could aid in immediately treating these residual cancer cells and potentially prevent or delay recurrence prior to adjuvant treatments being applied. Indeed, previous reports using LDDS loaded with gemcitabine [37] and TMZ [37] have demonstrated enhanced activity against GBM in this manner.

The cytotoxicity of Dox and Ola was next investigated against a panel of patient-derived and murine GBM cell lines (Table 1). Dox demonstrated high potency against all cell lines, with IC50 values of 0.04 ± 0.01, 0.05 ± 0.01 and 0.17 ± 0.01 µM calculated for SB28-Ohlfest, GIN28 and GCE28 cell lines, respectively. Ola exhibited higher IC50 values than Dox, with values of 9.5 ± 2.0, 24.6 ± 5.6 and 35.3 ± 5.2 µM calculated for SB28-Ohlfest, GIN28 and GCE28 cell lines, respectively, indicating lower potency than Dox. Ola was significantly more potent against SB28-Ohlfest cells compared to GIN28 (*p* < 0.05) or GCE28 (*p* < 0.001). The reason for these different susceptibilities to Ola treatment is unclear, but may be a result of altered mechanisms of DNA damage and/or DNA repair pathways, such as homologous repair [38, 39]; however, further experimentation is required to confirm this.

**Table 1:** Recorded IC50 values (mean ± SEM; *n* = 3) of Dox and Ola across a panel of GBM cell lines

	IC50 Value (µM)	
	Dox	Ola
SB28-Ohlfest	0.04 ± 0.01	9.47 ± 1.99
GIN28	0.05 ± 0.01	24.63 ± 5.57
GCE28	0.17 ± 0.01	35.30 ± 5.23

**Fig. 2:**
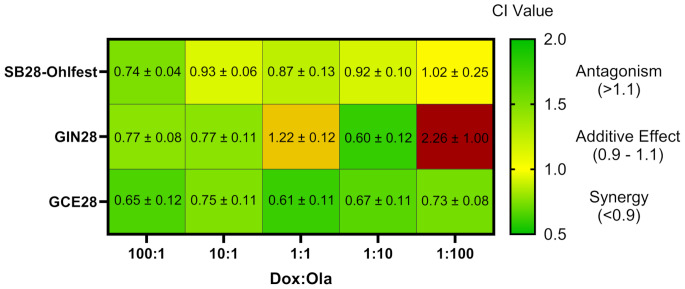
Dox/Ola combination is synergistic against GBM in vitro Combination Index (CI) values (mean ± SEM; *n* = 3) of Dox/Ola combinations at varying ratios using Chou and Talay method for quantification of synergy [31, 32] against a panel of GBM cell lines

The impact of the combination of Dox and Ola against the panel of GBM cell lines was investigated using a wide range of Dox: Ola ratios from 1:100 to 100:1. This range accounts for varying drug release profiles when co-loaded into the PELCLE HG, and also to reflect larger drug-drug concentration differences that may be experienced post-release due to differing drug distribution and tumour uptake levels within the brain microenvironment. Combination Index (CI) values were calculated using the Chou and Talay method [31, 32] with CI values < 0.9 deemed synergistic, values between 0.9 and 1.1 deemed additive and values >1.1 deemed antagonistic. Calculated values have been reported in Fig. 2, with a broadly synergistic effect noted at all ratios. This promising observation of synergy indicates that additional mechanisms of anti-cancer activity may be induced with the applied Dox: Ola ratios on GBM cells. From previous work in triple negative breast cancer cells, the synergy of Dox and Ola was attributed to the Ola-mediated inhibition of repair of DNA damage induced by Dox-mediated DNA intercalation and reactive oxygen species (ROS) generation, followed by the subsequent induction of apoptosis [23]. Future work is required to fully confirm these responses in GBM cells; however, considering the ubiquitous nature of PARP1 mediated DNA repair [40] and Dox-DNA intercalation, it is likely that these mechanisms are conserved.

**Fig. 3:**
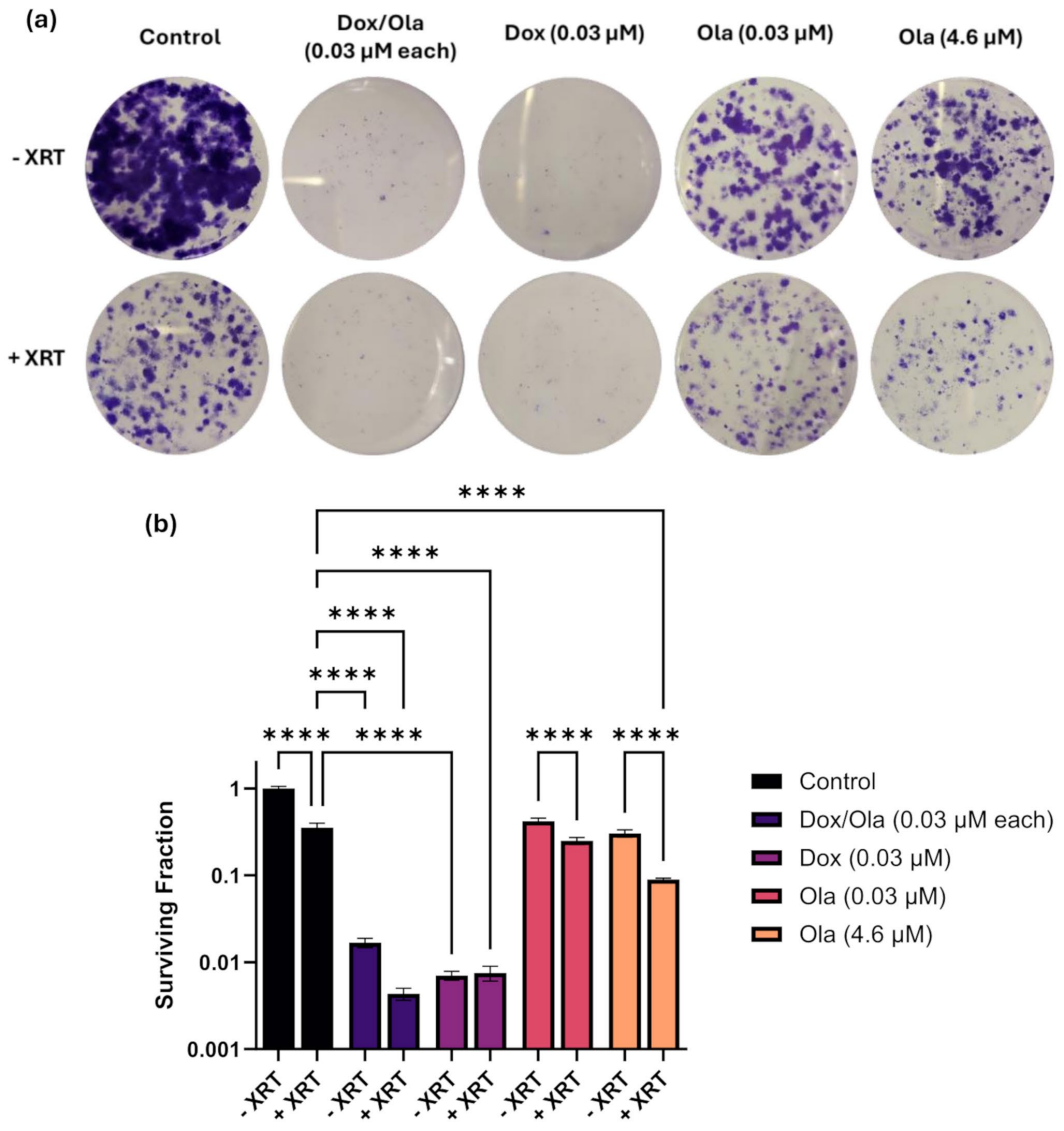
Assessment of Dox/Ola as single and combination treatment adjuvant to XRT in vitro (**a**) Clonogenic survival assay in SB28-Ohlfest cells, with (**b**) calculated survival fraction (mean ± SEM; *n* = 3). Statistical analysis was carried out using a two-way ANOVA with Tukey’s multiple comparisons, with differences considered significant when **** *p* < 0.0001, *** *p* < 0.001, ** *p* < 0.01, * *p* < 0.05. For ease of visualisation, significance is only reported between treatment groups ± XRT and between the control + XRT and all other treatment groups. Additional statistical significance has been reported in Table S1

A clonogenic survival assay was undertaken in SB28-Ohlfest cells to assess the impact of Dox and/or Ola in combination with radiation in vitro (Fig. 3a). For all treatment conditions (control ± 2 Gy, Dox/Ola at 0.03 µM each ± 2 Gy, Dox at 0.03 µM ± 2 Gy, Ola at 0.03 µM ± 2 Gy or Ola at 4.6 µM ± 2 Gy) the surviving fraction (SF) was calculated (Fig. 3b). The addition of XRT to the control group led to a significant decrease in SF (0.35 ± 0.04 and 1.00 ± 0.06 with and without XRT, respectively, *p* < 0.0001), indicating that XRT alone impacts the clonogenicity of this cell line. The addition of Dox, Ola or combinations of the two compounds with or without the addition of XRT, led to a significant decrease in SF when compared to the control group with the addition of XRT (*p* < 0.0001 when compared with Dox/Ola ± XRT, Dox ± XRT, and Ola at 4.6 µM + XRT), indicating the efficacy of these drugs in reducing the clonogenicity of this cell line.

The addition of Ola dosed at 0.03 µM to the cells resulted in an SF of 0.42 ± 0.03, which was significantly decreased with the addition of XRT (SF of 0.25 ± 0.02 recorded (*p* < 0.0001)). Similar results were observed at higher concentrations of Ola of 4.6 µM (0.09 ± 0.003 and 0.30 ± 0.03 with and without XRT, respectively, *p* < 0.0001), confirming the radiosensitising nature of Ola in SB28-Ohlfest cells. When Dox was applied as a single agent at 0.03 µM, no significant change in SF was observed with and without the addition of XRT (0.007 ± 0.001 and 0.007 ± 0.001), indicating a lack of radiosensitisation. Previous studies have reported radiosensitising activity with Dox in various cancer models, including lung cancer [41] and breast cancer [42]. Interestingly and consistent with our observations, a lack of sensitisation to radiotherapy has been observed with Dox in glioma cell lines [43, 44]. Nonetheless, the improvements in recorded SF values with the various treatment groups are encouraging and highlight the capabilities of these drug combinations with adjuvant XRT against this GBM cell line.

**Fig. 4:**
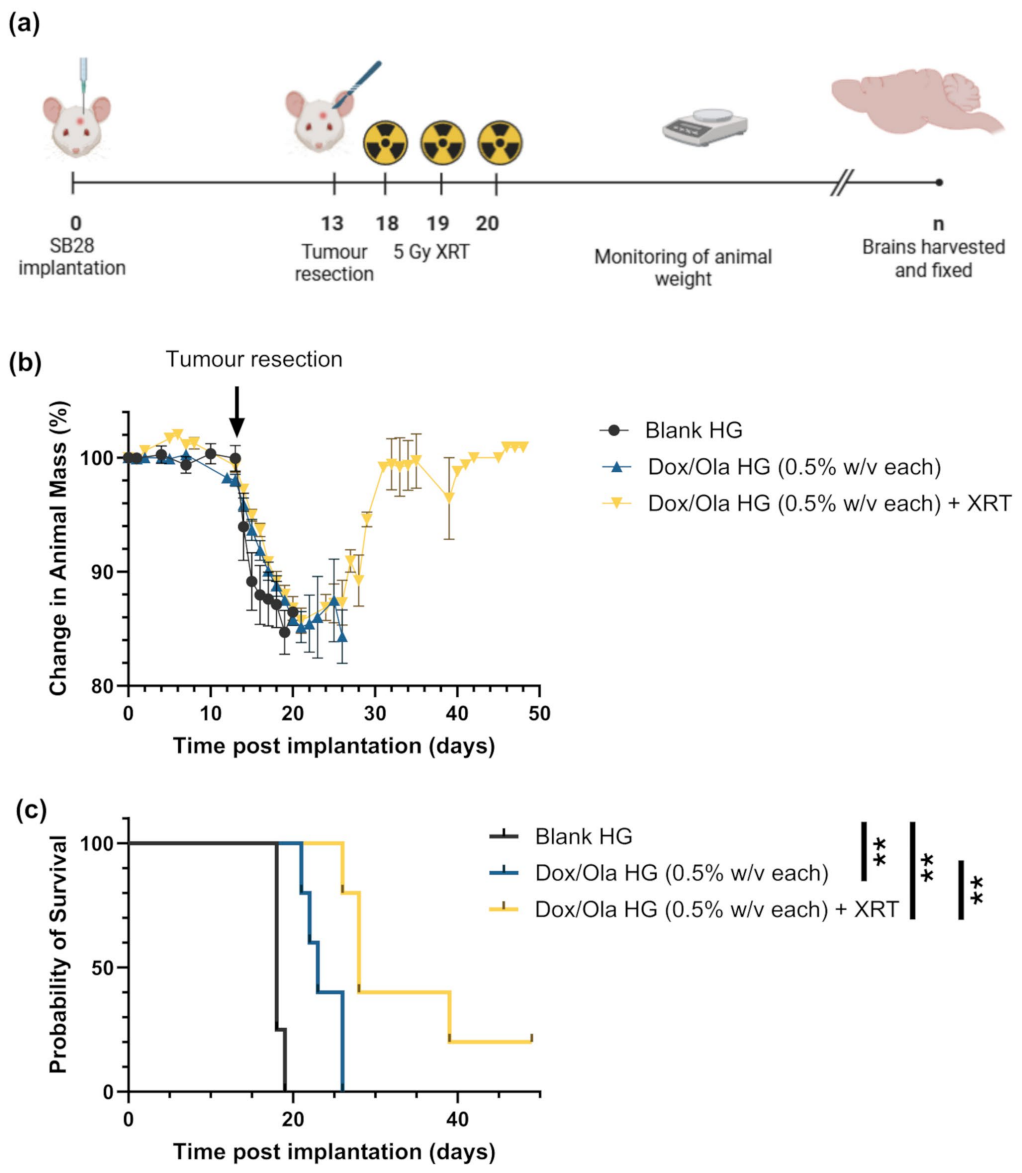
In vivo efficacy of locally delivered drug-loaded PELCLE HG in an orthotopic GBM model. (**a**) Schematic representation of the in vivo efficacy schedule. (**b**) Recorded average change (%) in body weight of mice implanted with SB28-Ohlfest GBM cells, which following surgical resection were treated with blank PELCLE HG (*n* = 4), or Dox/Ola loaded PELCLE HG at 0.5% (w/v) with (*n* = 5) or without (*n* = 5) the addition of 5 Gy XRT over the course of experiment (mean ± SEM), with the time of tumour resection indicated. (**c**) Kaplan-Meier overall survival plots of blank PELCLE HG (*n* = 4), or Dox/Ola loaded PELCLE HG at 0.5% (w/v) with (*n* = 5) or without (*n* = 5) the addition of 5 Gy XRT over the course of the experiment. Animals alive at termination of experiment after 49 days post tumour implantation were deemed LTS. Significance levels determined by the log-rank test, with ** representing *p* < 0.01

A pilot in vivo efficacy study of Dox/Ola loaded HGs was undertaken using a GBM mouse model to assess the feasibility of PELCLE HG application intraoperatively and to investigate the efficacy of Dox/Ola loaded PELCLE HGs, with and without the addition of XRT. The SB28-Ohlfest syngeneic mouse GBM model was chosen for this work as it is an invasive tumour model [45] that was developed to better mimic human GBM in animal models [46]. Mice implanted with SB28-Ohlfest cells underwent surgical resection of macroscopic tumour and were then exposed to either blank PELCLE HG (*n* = 4), or PELCLE HG loaded with Dox/Ola at 0.5% (w/v) of each drug with or without the addition of 5 Gy XRT (*n* = 5 for both treatments). The treatment schedule is indicated in Fig. 4a. Animal weight was monitored throughout the course of the experiment (Fig. 4b). An initial drop in weight was observed in the days following craniotomy and surgical resection of the tumours, which was not unexpected given the invasive nature of the surgery, but weights did not drop below 80% and animal weights recovered over the course of the experiment, particularly for the long-term survivor (LTS) in the Dox/Ola with XRT group. Additionally, no adverse effects were noted across all groups, indicating that all treatments were well tolerated.

A Kaplan-Meier survival plot is shown in Fig. 4c, where a significant increase in survival can be noted between the blank HG and the Dox/Ola loaded HG (*p* < 0.01), with recorded median survival of 18 and 23 days, respectively (Table 2). The addition of adjuvant XRT increased the median survival to 28 days, with a significant increase reported when compared to both the blank HG and the drug loaded HG without XRT (*p* < 0.01 in both cases). Furthermore, there was one LTS remaining at the end of the experiment.

**Table 2:** Summary of mean and median overall survival in GBM orthotopic resection model (SB28-Ohlfest) treated with blank or Dox/Ola loaded (0.5% w/v each) PELCLE HGs, with and without radiotherapy

Treatment Group	*n*	Mean survival (days)^a^	Median survival (days)	SEM	LTS	%LTS
Blank HG	4	18.3	18.0	0.3	0	0
Dox/Ola 0.5% (w/v) each	5	23.6	23.0	1.0	0	0
Dox/Ola 0.5% (w/v) each + XRT	5	34	28.0	4.4	1	20

^*a*^Estimation is limited to the longest survival time if censored

**Fig. 5:**
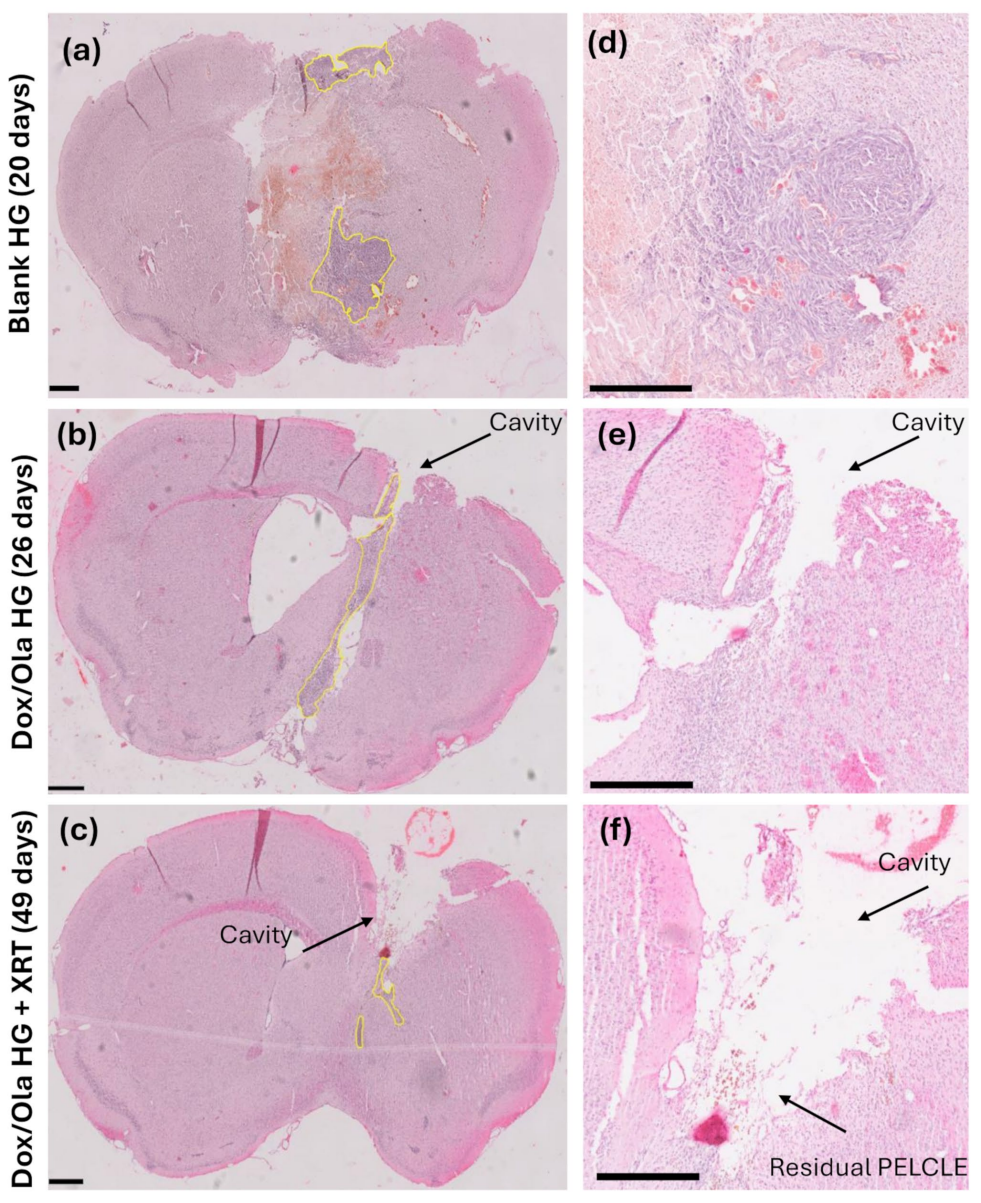
Histological assessment of longest-term survivor for control and Dox/Ola +/- XRT in vivo. Representative H&E staining of longest survivor in treatment groups receiving blank PELCLE HG, or Dox/Ola loaded PELCLE HG at 0.5% (w/v) with or without the addition of 5 Gy XRT. (**a**) – (**c**) Images taken at 1.25 X magnification, scale bar = 500 μm. The presence of recurrent tumour (in yellow) or resection cavity and residual PELCLE (black arrows) are indicated. (**d**) – (**f**) Images taken at 5 X magnification, scale bar = 500 μm

Histological evaluation of the brains from the longest survivors in each group was undertaken to investigate the tissue level effects of the treatments. H&E staining demonstrated the presence of a large, infiltrative tumour in the blank PELCLE HG treated animal (Fig. 5a and d), which is unsurprising given the invasive nature of this tumour (20 days survival following tumour implantation and 7 days survival following tumour resection and treatment). The administration of the Dox/Ola loaded PELCLE HG led to a reduction in bulk tumour size, but where tumour infiltration through parenchyma was still evident (Fig. 5b and e). When Dox/Ola loaded PELCLE HG was applied with adjuvant XRT, a large cavity was evident within the brain, with some residual PELCLE HG present at the bed of the cavity (Fig. 5c and f). While there are some small incidences of tumour present within the parenchyma (which is to be expected with such an invasive tumour), the absence of a large recurrent tumour is a promising indicator of the suitability of this treatment regime against this tumour type. Similar findings were noted from the histological evaluation of representative brains from animals which exhibited median survival in each group (Figure S3). A large tumour was evident in the blank PELCLE treated animal. Dox/Ola loaded PELCLE HG treatment resulted in a reduction in tumour size, with tumour size further reduction with the application of Dox/Ola loaded PELCLE HG and adjuvant XRT.

The improved survival and reduction in tumour recurrence following treatment with Dox/Ola loaded PELCLE HG with the addition of adjuvant XRT is in agreement with the recorded in vitro data reported in Fig. 3a and b, where a decrease in surviving fraction was noted when Dox/Ola was applied to SB28-Ohlfest cells in combination with XRT. The radiosensitising nature of Ola has been widely reported in the literature against several cancer types [47, 48] and while our results are not unexpected, it is promising to see the applicability of Ola against GBM cell lines in vivo. Additionally, Ola has undergone several clinical trials in combination with other chemotherapeutic agents which were shown to be well tolerated and efficacious [49, 50], setting a precedent for our LDDS system loaded with Dox/Ola against GBM to be investigated further.

## Conclusions

In this work, we have demonstrated the applicability and efficacy of LDDS loaded with Dox and Ola against GBM. The thermoresponsive properties and the injectability of the drug loaded system are advantageous for application in a tumour resection model due to the ability of the gel to conform to the shape of the cavity when set, improving persistent contact with the surrounding parenchyma. The sustained release of Dox and Ola from the HG is suitable to bridge the oncological treatment gap between surgical resection and administration of chemo/radiotherapy for treatment of GBM in the clinic. The two drugs have been demonstrated to work synergistically at a range of ratios against a panel of GBM cell lines in vitro, and importantly, the radiosensitising nature of Ola results in a significant increase in efficacy against the SB28-Ohlfest cell line when adjuvant XRT is applied. In an in vivo GBM resection model, Dox/Ola HGs exhibited enhanced survival compared to blank HG, and additionally, when adjuvant XRT was applied, median survival was further increased, and a long-term survivor remained at the termination of the study. The potentiated response to the combined local delivery of Dox/Ola and XRT is promising for future treatments of GBM.

## Supplementary Information

Below is the link to the electronic supplementary material.

Supplementary Material 1

## Data Availability

All relevant data are available on request via the University of Nottingham or ruman.rahman@nottingham.ac.uk.
