# Supplementary material for: Local delivery of Doxorubicin and Olaparib loaded injectable hydrogels with adjuvant radiotherapy improves survival in a glioblastoma in vivo model
Source: Drug Deliv Transl Res. 2025 Oct 23;16(7):2367–81. doi: 10.1007/s13346-025-02003-7 (PMC13294248; doi:10.1007/s13346-025-02003-7)
Supplement: Supplementary file 1 — Supplementary Material 1 [file 13346_2025_2003_MOESM1_ESM.docx]

**Local Delivery of Doxorubicin and Olaparib Loaded Injectable Hydrogels with Adjuvant Radiotherapy Improves Survival in a Glioblastoma *in vivo* Model**

Robert Cavanagh^a^, Gary Shaw^b^, Phoebe McCrorie^c^, Amr ElSherbenny^a^, Alina Pandele^c^ Supisara Jearranaiprepame^a^, Bayan Ghanem^a^, Natalie Allcock^d^, Heiko Wurdak^b^, Ryan K Mathew^b,e^, Cameron Alexander^a^, Ruman Rahman^c^, Cara Moloney^a,c^

^a^ School of Pharmacy, University of Nottingham, NG7 2RD, UK.

^b^ Leeds Institute of Medical Research, School of Medicine, University of Leeds , Leeds, LS9 7TF, UK.

^c^ School of Medicine, Biodiscovery Institute, University of Nottingham, NG7 2RD, UK.

^d^ Electron Microscopy Facility, University of Leicester, LE1 7HB, UK.

^e^ Department of Neurosurgery, Leeds Teaching Hospitals NHS Trust, Leeds, LS1 3EX,UK

^1^H NMRs with full structure assignment for the mPEG-PLA, mPEG-PLA-PCL and PELCLE have been reported by us previously [1]. Here we report an adapted version of these spectra, highlighting the key peaks which confirm the successful addition of the various blocks to the polymeric structure (**Figure S1**). Additional characterisation data, including GPC and FTIR can be found in our previous report [1].

**Figure S1.** ^1^H NMR spectra of mPEG-PLA, mPEG-PLA-PCL and PELCLE (mPEG-PLA-PCL-HMDI-PCL-PLA-mPEG), adapted from a previous report [1] with key features identified in each spectrum.

**Figure S2.** Cumulative % release of **(a)** Dox and **(b)** Ola from PELCLE HG prepared at 30% (w/v) and loaded with Dox, Ola or a combination at loadings of 0.5 % (w/v) of each drug.

**Table S1.** Full statistical analysis of calculated survival fraction following clonogenic survival assay of SB28-Ohlfest cells. Analysis was carried out using a two-way ANOVA with Tukeys multiple comparisons, with differences considered significant when **** p <0.0001, *** p < 0.0002, ** p < 0.002, * p < 0.0332.

| **Group 1** | **Group 2** | **Summary** | **p value** |
| --- | --- | --- | --- |
| Control - XRT | Dox/Ola (0.03 µM each) - XRT | **** | <0.0001 |
| Control - XRT | Dox (0.03 µM) - XRT | **** | <0.0001 |
| Control - XRT | Ola (0.03 µM) - XRT | **** | <0.0001 |
| Control - XRT | Ola (4.6 µM) - XRT | **** | <0.0001 |
| Control - XRT | Control + XRT | **** | <0.0001 |
| Control - XRT | Dox/Ola (0.03 µM each) + XRT | **** | <0.0001 |
| Control - XRT | Dox (0.03 µM) + XRT | **** | <0.0001 |
| Control - XRT | Ola (0.03 µM) + XRT | **** | <0.0001 |
| Control - XRT | Ola (4.6 µM) + XRT | **** | <0.0001 |
| Dox/Ola (0.03 µM each) - XRT | Dox (0.03 µM) - XRT | ns | >0.9999 |
| Dox/Ola (0.03 µM each) - XRT | Ola (0.03 µM) - XRT | **** | <0.0001 |
| Dox/Ola (0.03 µM each) - XRT | Ola (4.6 µM) - XRT | **** | <0.0001 |
| Dox/Ola (0.03 µM each) - XRT | Control + XRT | **** | <0.0001 |
| Dox/Ola (0.03 µM each) - XRT | Dox/Ola (0.03 µM each) + XRT | ns | >0.9999 |
| Dox/Ola (0.03 µM each) - XRT | Dox (0.03 µM) + XRT | ns | >0.9999 |
| Dox/Ola (0.03 µM each) - XRT | Ola (0.03 µM) + XRT | **** | <0.0001 |
| Dox/Ola (0.03 µM each) - XRT | Ola (4.6 µM) + XRT | ns | 0.0997 |
| Dox (0.03 µM) - XRT | Ola (0.03 µM) - XRT | **** | <0.0001 |
| Dox (0.03 µM) - XRT | Ola (4.6 µM) - XRT | **** | <0.0001 |
| Dox (0.03 µM) - XRT | Control + XRT | **** | <0.0001 |
| Dox (0.03 µM) - XRT | Dox/Ola (0.03 µM each) + XRT | ns | >0.9999 |
| Dox (0.03 µM) - XRT | Dox (0.03 µM) + XRT | ns | >0.9999 |
| Dox (0.03 µM) - XRT | Ola (0.03 µM) + XRT | **** | <0.0001 |
| Dox (0.03 µM) - XRT | Ola (4.6 µM) + XRT | ns | 0.0685 |
| Ola (0.03 µM) - XRT | Ola (4.6 µM) - XRT | * | 0.0241 |
| Ola (0.03 µM) - XRT | Control + XRT | ns | 0.6870 |
| Ola (0.03 µM) - XRT | Dox/Ola (0.03 µM each) + XRT | **** | <0.0001 |
| Ola (0.03 µM) - XRT | Dox (0.03 µM) + XRT | **** | <0.0001 |
| Ola (0.03 µM) - XRT | Ola (0.03 µM) + XRT | **** | <0.0001 |
| Ola (0.03 µM) - XRT | Ola (4.6 µM) + XRT | **** | <0.0001 |
| Ola (4.6 µM) - XRT | Control + XRT | ns | 0.9107 |
| Ola (4.6 µM) - XRT | Dox/Ola (0.03 µM each) + XRT | **** | <0.0001 |
| Ola (4.6 µM) - XRT | Dox (0.03 µM) + XRT | **** | <0.0001 |
| Ola (4.6 µM) - XRT | Ola (0.03 µM) + XRT | ns | 0.7828 |
| Ola (4.6 µM) - XRT | Ola (4.6 µM) + XRT | **** | <0.0001 |
| Control + XRT | Dox/Ola (0.03 µM each) + XRT | **** | <0.0001 |
| Control + XRT | Dox (0.03 µM) + XRT | **** | <0.0001 |
| Control + XRT | Ola (0.03 µM) + XRT | ns | 0.0772 |
| Control + XRT | Ola (4.6 µM) + XRT | **** | <0.0001 |
| Dox/Ola (0.03 µM each) + XRT | Dox (0.03 µM) + XRT | ns | >0.9999 |
| Dox/Ola (0.03 µM each) + XRT | Ola (0.03 µM) + XRT | **** | <0.0001 |
| Dox/Ola (0.03 µM each) + XRT | Ola (4.6 µM) + XRT | ns | 0.0939 |
| Dox (0.03 µM) + XRT | Ola (0.03 µM) + XRT | **** | <0.0001 |
| Dox (0.03 µM) + XRT | Ola (4.6 µM) + XRT | ns | 0.1018 |
| Ola (0.03 µM) + XRT | Ola (4.6 µM) + XRT | **** | <0.0001 |

**Figure S3.** Representative H&E staining of animals exhibiting median survival in treatment groups receiving blank PELCLE HG, or Dox/Ola loaded PELCLE HG at 0.5% (w/v) with or without the addition of 5 Gy XRT. **(a) – (c)** Images taken at 1.25 X magnification, scale bar = 500 µm.

[1] A. Elsherbeny, H. Bayraktutan, N. Gumus, P. Mccrorie, A. Garcia-Sampedro, S. Parmar, A.A. Ritchie, M. Meakin, U.C. Oz, R. Rahman, J.C. Ashworth, A.M. Grabowska, C. Moloney, C. Alexander, Pentablock thermoresponsive hydrogels for chemotherapeutic delivery in a pancreatic cancer model, Biomater Sci-Uk 13(7) (2025) 1831-1848.
